# Articular Cartilage Regeneration in Osteoarthritis

**DOI:** 10.3390/cells8111305

**Published:** 2019-10-23

**Authors:** Livia Roseti, Giovanna Desando, Carola Cavallo, Mauro Petretta, Brunella Grigolo

**Affiliations:** 1 Laboratorio RAMSES, IRCCS Istituto Ortopedico Rizzoli, Via di Barbiano 1/10, 40136 Bologna, Italy; livia.roseti@ior.it (L.R.); giovanna.desando@ior.it (G.D.); mauro.petretta@ior.it (M.P.); brunella.grigolo@ior.it (B.G.); 2 RegenHU LTD, Z.I. Du Vivier 22, CH-1690 Villaz-St-Pierre, Switzerland

**Keywords:** osteoarthritis, cartilage, cell-based therapy, tissue-based therapy, gene therapy

## Abstract

There has been considerable advancement over the last few years in the treatment of osteoarthritis, common chronic disease and a major cause of disability in older adults. In this pathology, the entire joint is involved and the regeneration of articular cartilage still remains one of the main challenges, particularly in an actively inflammatory environment. The recent strategies for osteoarthritis treatment are based on the use of different therapeutic solutions such as cell and gene therapies and tissue engineering. In this review, we provide an overview of current regenerative strategies highlighting the pros and cons, challenges and opportunities, and we try to identify areas where future work should be focused in order to advance this field.

## 1. Articular Cartilage in Osteoarthritis

The pathologic traits of osteoarthritis (OA) consist of articular cartilage degradation together with subchondral bone thickening, osteophyte formation, synovial inflammation, ligament degeneration, and capsule hypertrophy [1]. OA is a common chronic joint disease characterized by pain, deformity, instability, and reduction of motion and function [2]. Unlike focal defects which, in general, involve a younger population who suffered an acute trauma and require localized treatment, OA lesions affect elderly patients and often the whole joint surface [3]. OA is indeed one of the main causes of disability in older adults, affecting about 10% of men and 18% of women over the age of sixty. The universal increase in life expectancy makes OA one of the most important causes of disability [3]. The pathology mainly involves knees, hips, cervical and lumbosacral spine, and ankle. The distal, proximal inter-phalangeal, and carpometacarpal joints may be affected as well. Symptoms include pain with gradual development which is worsened or triggered by activity, stiffness on waking and after inactivity, and joint swelling [2,3,4]. Although still not completely elucidated, the aetiology is considered multifactorial with genetic, constitutional, and environmental components [2]. OA diagnosis occurs through standard X-rays of the most symptomatic joints which generally reveal marginal osteophytes, articular space narrowing, increased density of subchondral bone, subchondral cysts formation, bone remodeling and effusion [3]. OA is classified into primary or idiopathic and secondary due to trauma, congenital and metabolic defects, infections, endocrine and neuropathic diseases, disorders altering the normal structure and function of articular cartilage, and intense or incongruous work or sport activities. The prevalent risk factors include age, gender, previous joint injuries, obesity, genetic predisposition, and mechanical factors [4].

**Figure 1:**
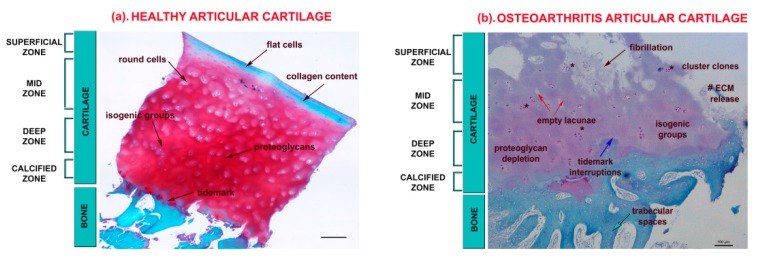
Representative micrographs of articular cartilage tissues representing healthy (**a**) and OA (**b**) stained with Safranin-O/Fast Green staining taken from the laboratory archive. Representation of typical healthy and OA features in the superficial, mid, deep, and calcified zones of articular cartilage. Scale bar: 100 µm; red staining: proteoglycan content; pinkish staining: depletion of proteoglycan content; green staining: collagen content. (**a**) Healthy articular cartilage, black arrows report: (i) the presence of flat cells in the ECM rich of collagen fibres in the superficial zone; (ii) the presence of round cells in the ECM rich of proteoglycans in the mid-zone; (iii) the presence of isogenic groups in the ECM rich of proteoglycans in the deep zone; and (iv) the presence of tidemark in the calcified zone. (**b**) OA articular cartilage, black arrow reports the presence of fibrillation in the superficial zone; red arrows report the presence of empty lacunae; blue arrow: tidemark interruptions; green arrow: trabecular spaces; # reports ECM release; * cluster clones.

Cartilage alterations in OA mainly concern an imbalance in tissue remodeling due to changes in chondrocyte behaviour [5]. Adult articular, hyaline cartilage is a viscoelastic, avascular connective tissue with a lubricated surface having friction-reducing and load-bearing functions [1]. It is composed of a structured network of dense extracellular matrix (ECM) containing highly differentiated cells, termed chondrocytes, with low metabolic activity and surviving under hypoxic conditions (<5% pO_2_). Water, collagens (mainly type II collagen fibres), large aggregates of proteoglycans (principally aggrecan) and other non-collagenous proteins (i.e., link protein, fibronectin, and cartilage oligomeric matrix protein (COMP)) are the predominant components of the ECM [6,7]. In such a network, chondrocytes are regularly distributed in lacunae (containing one chondrocyte or, when it divides, more cells forming isogenous groups) in four different zones—superficial, middle, deep and calcified—which are responsible for tissue homeostasis (Figure 1). Chondrocytes in the superficial zone are small and flat and surrounded by abundant collagen fibres whose content gradually decreases along the thickness, while proteoglycans increase; the mid-zone contains round chondrocytes; the deep zone displays chondrocytes oriented in vertical columns perpendicular to the surface [6,7].

Generally, OA articular surface displays swelling which progresses with fibrillation and finally to full-thickness erosions that expose the subchondral bone [2]. Chondrocytes become “activated” by producing ECM-degrading enzymes such as matrix metalloproteinases (MMPs) and MMPs with thrombospondin-like motifs (ADAMTS). In particular, MMP-13 plays a key role in the degradation of collagen type II, while ADAMTS-4 and -5 both act on aggrecan [8,9]. To this end, such enzymes contribute to modulate the expression of several cytokines, chemokines, inflammatory mediators and matrix-degrading enzymes by activating several signaling pathways including Notch and nuclear factor kappa-light-chain-enhancer of activated B cells (NFKB) [9], and deregulating the expression levels of some MicroRNAs (miRNAs) (endogenous small non-coding RNAs that suppresses gene expression by binding to complementary segments of messenger RNA and interfering with the formation of proteins by translation) [8,10,11]. The progressive loss of cartilage structural architecture, together with an increased osteoclast activity in the subchondral bone, causes interruptions in the tidemark with the presence of bony channels carrying inflammatory cells and blood vessels. In this way, the resistance of normal articular cartilage to neovascularization is overcome, as demonstrated also by the production of pro-angiogenic factors such as Vascular Endothelial growth factors (VEGF) [12]. Another key feature of OA is the presence of clonal clusters due to the increased proliferation activity by chondrocytes that produce inflammatory mediators, such as cytokines including interleukin 1β (IL-1β), interleukin 6 (IL-6), tumour necrosis factor-α (TNF-α) reactive oxygen species (ROS) and nitric oxide (NO), all contributing and accelerating the degradation and triggering apoptosis processes (presence of empty lacunae and positivity for caspases mediators). Finally, chondrocytes tend to differentiate towards an hypertrophy-like phenotype (enlarged cytoplasm mainly positive for type X collagen and MMP-13) and start to deposit calcium in the ECM as occurs in the endochondral ossification process in the epiphyseal plate [13,14]. Along with articular cartilage alterations, subchondral bone undergoes sclerosis with thickening of the subchondral plate, osteophytes, and cysts formation (Figure 1). In general, osteochondral alterations are often accompanied by a high amount of key biochemical markers of tissue breakdown and inflammation, including pro-collagen pro-peptide of type I and II collagen (PINP; PIINP), carboxy-terminal pro-collagen pro-peptide of type I and II collagen (PICP; PIICP), C-terminal cross-linking telopeptides (CTX-II), osteocalcin (OC), urinary total pyridinoline (PYD), and bone sialoprotein (BSP) [15].

## 2. Osteoarthritis Treatments

Despite the high prevalence and morbidity of OA, there is a current lack of an effective treatment. Being a multifactorial disease, it is likely that a multifactorial approach is needed [3]. The goals of curing OA are to slow down the progression of the early forms and to relieve pain and preserve as much as possible joint mobility and function in the late stages [16]. Current treatments can be divided into non-surgical and surgical. Non-surgical treatments include: physical rehabilitation; support devices; exercises to maintain muscle strength, body flexibility and resistance; patient education for a change in lifestyle; in the obese, weight reduction is important. Additional therapies comprise drug therapy and surgery [17,18]. Among drugs, paracetamol is the first choice for pain treatment. For patients who do not respond adequately, non-steroidal anti-inflammatory drugs (NSAIDs) and, more recent, COX-2 inhibitors (an enzyme necessary for prostaglandin synthesis) should be considered, these last having the advantage of causing less toxicity [19]. Topical application of creams (e.g., capsaicin, NSAIDs) can be useful both as monotherapy and in combination with oral analgesics. Dietary supplements (such as glucosamine sulfate and chondroitin-sulfate) have been tested too. Opioid administration is an option, but prolonged use often causes physical dependence. Intra-articular injections of corticosteroids can relieve pain; the use of hyaluronic acid (HA) as visco-supplementation can restore normal joint lubrication [3]. Conventional and biologic DMARDs, like a monoclonal antibody to TNFα, are under investigation [20]. Surgical treatments, offered to symptomatic patients when the previous therapies are ineffective, can be conservative or radical [3]. In conservative procedures, usually for young patients presenting early-stage lesions, the damaged tissues are left in place. Arthroscopic lavage and cartilage debridement should relieve symptoms by removing the debris and inflammatory cytokines [21,22]. Arthrodesis, which fuses joints in a permanent position, can be applied to increase strength and reduce pain. Osteotomy involves adding or removing a small section of bone either above or below the joint; it is aimed at unloading the damaged area, reducing pain, and slowing the degenerative process [21,22]. “Marrow stimulation techniques”, like subchondral drilling, consist in subchondral bone penetration, thus inducing bleeding and migration of bone marrow stem cells to the site of injury, along with blood clot formation [23]. However, generally, the resulting repair tissue is mainly composed of fibrocartilage [24]. In total joint arthroplasty, currently recommended for elderly patients suffering from end-stage lesions, the articular tissue is replaced by an artificial endoprosthesis [21,22]. Although this therapeutic option has been shown to relieve pain and improve mobility in people, it may carry several complications like stiffness, instability, aseptic loosening, infection, prosthesis failure, and mal-alignment [21,22].

## 3. Regenerative Medicine as a Novel Therapeutic Option

In the last few years, regenerative medicine strategies have been developed as an alternative to traditional surgical methods, having the ambitious aim to create a new tissue displaying the most similar features to native cartilage [25]. Tissue composition and architecture is deeply connected with function, thus the ability to re-create structure is believed to be essential for regeneration [26]. Regenerative medicine deals with the development of innovative therapies focused on the repair, regeneration and replacement of cells, tissues or organs in order to restore structure and physiological functions compromised by diseases, trauma, congenital defects, or aging [26]. The regeneration of articular cartilage tissue still remains one of the main challenges in the orthopaedics field [19]. The problem arises from its avascularity that limits progenitor cells infiltration and thus the repair process to occur. As regards toosteochondral damages also involving the subchondral bone, a repair process is initiated by undifferentiated mesenchymal stem/stromal cells (MSCs) from the bone marrow, but the resulting tissue is fibrocartilage, which is a poor substitute for hyaline articular cartilage [24]. In addition, the actively inflammatory environment of the injured joint has a role in influencing the repair potential [27]. The osteochondral grafting regenerative approach consists of the replacement of the damaged osteochondral area with autologous (mosaicoplasty) or allogeneic (allograft transplantation) (cartilage and bone) tissue. The technique may present problems due to donor site morbidity and graft failure in the autologous procedure or possible disease transmission and short cell viability in the allogeneic one [24].

**Figure 2:**
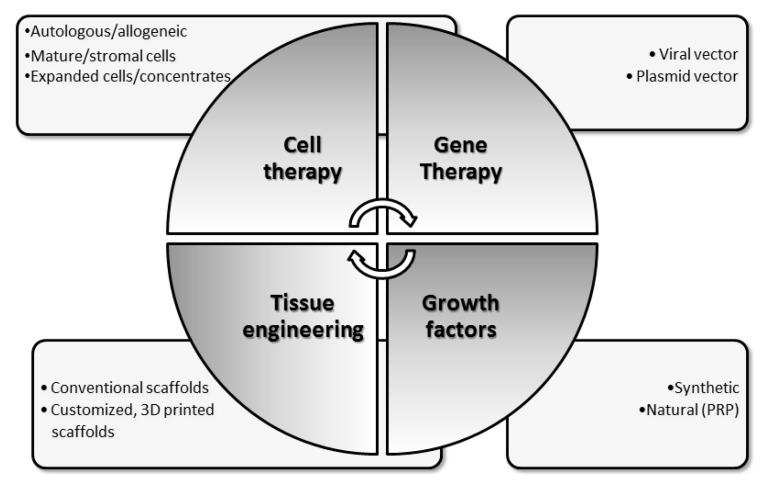
Graphical representation of regenerative medicine strategies utilized to treat OA. Clockwise from the upper left: cell therapy, i.e., injection, grafting or implantation of cells previously manipulated ex vivo or non-manipulated (concentrates); tissue engineering, relying on the use of scaffolds which act as a template for cell attachment and reproduce as closely as possible cartilage ECM and can be produced by conventional or additive manufacturing procedures; growth factors, synthetic on naturals, to be used as adjuvants to favor tissue regeneration; gene therapy, with the aim to treat human diseases by correcting an existing abnormality or providing a new function by means of viral and non-viral (plasmids) vectors.

Most recently developed regeneration strategies use a combination of several technologies that led to the development of different therapeutic solutions and methodologies: cell therapy; tissue engineering; gene therapy [26] (Figure 2).

A cell therapy is a clinical treatment consisting of the injection, grafting or implantation of cells previously manipulated ex vivo [28]. Cell administrations can be local or systemic, single or multiple. Cell sources may be autologous avoiding immune response issues and disease transmission or allogeneic (from living or cadaver donors) to eliminate donor-site morbidity and to maximize availability [24]. Cells can be expanded in culture or not (concentrates). The choice of a cell population that best allows reaching cartilage regeneration is still a challenge. In particular, an ideal cell component should be: viable; available and accessible; non-immunogenic; non-tumorigenic; phenotypically stable; responsive to bioactive factors [29]. The emerging of tissue engineering has given great hope in the scientific and medical field and its principles have also been used for the treatment of cartilage lesions [26]. It relies on the use of scaffolds which act not only as a template for cell attachment but that are also designed in order to reproduce as closely as possible cartilage ECM structure and thus to provide the appropriate environment for cell growth and chondrogenic differentiation. Moreover, they allow a more stable spatial distribution of cell, avoiding their dispersion in the articular space [30]. Scaffolds may differ for origin, composition, structure and status, but they should be able to carry the implantable cells maintaining their phenotype and try to mimic, as closely as possible, cartilage ECM [31]. Moreover, they should be biocompatible, biodegradable, not elicit an immune response, be bio-mimetic in order to induce chondrogenic differentiation and ECM production, display an architectural structure allowing cell colonization and exchange of nutrients, and possess mechanical properties to support tissue growth under native mechanical loads [32]. Both cell therapy and tissue engineering may be potentiated in their effects by the use of growth factors known to enhance the regeneration process and some therapeutic solutions have been developed in this direction [24]. Recent years have seen an increase in OA genomic studies. It is becoming apparent that many genes, each with small effect size, contribute to the risk, development, and progression of the disease. The multi-factorial origin and the localized nature of OA make it an ideal candidate for gene therapy [33]. Gene therapy is the process of introducing genomic material (trans-gene) in specific target cells, with the aim to treat human diseases by correcting an existing abnormality or providing a new function [34]. A carrier, termed expression vector, is genetically engineered to deliver the gene into the cells. There are two main classes of vectors: non-viral and viral. Non-viral vectors are commonly plasmids that can be transferred to recipient cells by physical or chemical methods. Viral vectors are adenoviruses, recombinant adeno-associated viral (rAAV), retroviruses, and baculoviruses. Non-viral methods are secure, easy to perform, and cost-effective, but delivery is less efficient than viral vectors [34].

### 3.1. Autologous Chondrocyte Implantation

Chondrocytes were the initial choice for cartilage tissue engineering applications since they are found in the native tissue. Autologous chondrocyte implantation (ACI) was first used for the treatment of knee cartilage defects by a Swedish group in 1994 [35]. The procedure consisted in the removal of small biopsies of cartilage (200/300 mg) from less loaded areas of the knee. Isolated chondrocytes were cultured in monolayer and, after 2–3 weeks, harvested as a suspension for implantation. The suspension was then injected into the defect under a periosteal flap derived from the proximal medial tibia (first-generation ACI) or, lately, under a bioengineered bilayer collagen membrane (second-generation ACI). ACI has become an established cell therapy and is widely applied as an effective solution for the treatment of lesions of cartilage in younger patients with symptoms of joint pain and swelling [36]. OA has been considered a contraindication for ACI since the degenerative microenvironment may cause the implanted chondrocytes to undergo undesired dedifferentiation or apoptosis, therefore undermining efficacy [19,30]. However, generally, studies on ACI recruited also patients with cartilage early degenerative changes showing outcomes similar to patients with injuries due to trauma [37]. For this reason Minas et al. conducted a trial where ACI was applied to early OA lesions. The results highlighted a reduction in pain and an improvement in function, hypothesizing a possible use of ACI for OA patients [37].

Tissue engineering has represented a strategic approach to overcome ACI complications such as periosteum flap hypertrophy, delamination, calcification or shrinkage, and cell leaking in the articular environment. Moreover, the monolayer in vitro expansion causes the loss of chondrocyte features, leading to a shift to a fibroblast-like phenotype. An important added value was provided by the possibility to perform implantation either by arthroscopy or by a small incision, rendering the procedure less invasive [38,39]. However, other issues are associated with the procedure itself, that is considered long, complicated, and expensive. In fact, a two-step intervention is required—the biopsy harvest and the implantation of the chondrocyte suspension—with the need for two hospitalization events and an increased risk for patients. In addition, the expansion phase is long-lasting and must be conducted strictly following the requirements of good manufacturing practice (GMP) [40], which are mandatory rules defining medicinal product quality, safety and efficacy [41]. Third generation ACI, also referred to as matrix-induced ACI (MACI), comprises the seeding of in vitro expanded chondrocytes onto a three-dimensional (3D) scaffold [42]. The engineered tissue is subsequently trimmed to the size of the defect and implanted with fibrin glue fixation. The tissue engineering approach, varying in terms of the scaffold utilized (i.e., collagen, HA; membrane, sponge, fibres), showed clinically and statistically significant improvement and no remarkable adverse events or safety issues [42]. Advantages include avoiding the need for a periosteal graft or fixation sutures and the possibility to perform implantation arthroscopically or with a small incision. The results obtained with ACI or MACI approaches may have encouraged studies on OA patients. To our knowledge, no clinical trials have been conducted except for Minas et al. [37].

### 3.2. Stem/Stromal Cell-Based Therapy

Despite favourable clinical results, scaffold-based ACI still displays limitations such as the scarce availability of the cell source, donor-site morbidity, and chondrocyte phenotypic instability in culture [38,41]. Therefore, cell types other than chondrocytes have been explored and, in particular, stem cells of different origins [43,44]. Several clinical trials have been conducted in the last years, but the heterogeneity of cell source, methodologies, and outcome measurement have hindered the possibility to fully evaluate the safety and efficacy of stem cells utilized for OA treatment [45].

Khalifeh et al. reported a double-blind, placebo-controlled clinical trial where 20 patients with knee OA received a single intra-articular injection of allogeneic placenta-derived stem cells. The control group was treated with saline solution. Chondral thickness improved in the cell-treated group at 24 weeks follow-up and no serious adverse effects were observed [46,47]. Park et al. administered allogeneic human umbilical cord blood-derived stem cells embedded in an HA hydrogel in 7 OA patients. During a 7-year follow-up, safety, the visual analogue scale (VAS) score, the International Knee Documentation Committee (IKDC) subjective score and magnetic resonance imaging (MRI) findings showed improvement. The histological findings at 1 year highlighted the formation of hyaline-like cartilage areas. Only mild to moderate adverse events were observed. There were no cases of osteogenesis or tumorigenesis [48]. In the study by Wang et al., patients with moderate or severe knee OA were treated with two intra-articular injections of human umbilical cord-derived stem cells. Sodium hyaluronate was used in the control group. Lysholm, Western Ontario and McMaster Universities Osteoarthritis Index (WOMAC) and Short Form (36) Health Survey (SF-36) evaluation systems highlighted a significant improvement in joint function and quality of life in the MSC-treated group at 6 months [49]. The regenerative potential of induced pluripotent stem cells (iPSCs) is gaining increasing attention since they have shown the ability to differentiate into chondrocytes [50]. However, several issues still remain to be solved such as potential genomic modifications and teratogenesis [24].

Adult MSCs have elicited significant interest in regenerative medicine since they have been identified in many organs and tissues, are easy to isolate, and do not imply ethical problems [44].

MSCS from bone marrow were the first type considered suitable for regenerative treatments. They were initially characterized for their ability to adhere to the surface of plastic plates with a fibroblast-like morphology, to proliferate in monolayer culture, and to maintain multipotency in vitro, including chondrogenesis. Recently, it was debated if the regeneration process would occur by means of implanted MSCs differentiation or their endocrine and paracrine activity on host cells or both [29]. Subsequent discoveries highlighted that exogenously supplied MSCs secrete in the sites of injury soluble growth factors and cytokines that exert immunomodulatory, anti-inflammatory and trophic (regenerative) effects on the patient’s own resident stem cells that form the new tissue [51,52]. Some reviews have already evaluated MSC-based clinical trials, generally observing that being MSCs in limited number within the bone marrow, culture expansion was necessary to reach amounts useful to treat larger defects or to support multiple administrations [3,44,53,54]. In several studies, patients with knee OA were injected with ex-vivo expanded autologous bone marrow MSCs. Outcomes generally improved in terms of pain, range of motion and physical examination, as highlighted by algo-functional indices (SF-36, IKDC, Lysholm and 0.64 Tegner scores) and no or mild transitory adverse events were observed [55,56,57,58,59,60]. Some trials were conducted by associating MSCs to HA-based scaffolds. The algo-functional indices and MRI imaging scans showed improvements respect to the control groups treated with HA alone [61,62]. Lamo-Espinosa et al., tested two MSCs doses—1.0 × 10^7^ and 1.00 × 10^8^—and found no clinical differences after a follow up of 4 years [62]. A few studies administered allogeneic bone marrow MSCs by knee intra-articular injection, observing improvement in terms of pain and function [63,64]. In particular, Gupta et al. tested different doses of pooled MSCs (25, 50, 75, or 150 × 10^6^ cells), followed by HA. A trend towards improvement was seen in the 25 × 10^6^ cells-dose group, although not statistically significant differences with respect to placebo control [64].

Lately, stem cells derived from subcutaneous adipose tissue (ASCs or ADSCs) have been considered suitable for application in regenerative therapies as well, since they appear similar to MSCs, in terms of characteristics and functions [65]. The advantage over MSCs is that ASCs are present in a higher percentage (5% compared to 0.01%) in the source tissue and can be harvested by a less invasive procedure, implying better patient compliance and lower costs [66,67]. Moreover, ASCs possess a protective effect towards MSCs [68]. In the work by Lu et al., a single intra-articular injection of ASCs was administered in the knee of OA patients, with a significant improvement of the WOMAC score and no serious adverse events during the follow-up period [69]. Other studies compared groups treated with different doses of ASCs. Although different, in general, three escalating doses were utilized. The best results were obtained in the high-dose group as indicated by the WOMAC score and defect size reduction, without adverse events (follow-up six months) [70,71,72] Similar results were obtained for shorter (three months) [73] and longer (twenty-four months [72]) follow up.

**Figure 3:**
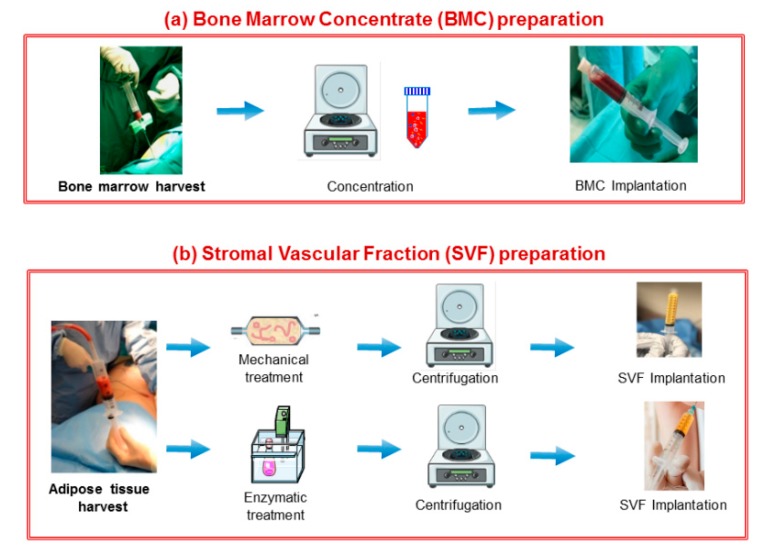
Schematic representation of the procedures needed to obtain (**a**) bone marrow aspirate (BMC) through a cell concentration step, from bone marrow; (**b**) stromal vascular fraction (SVF) from adipose tissue (enzymatic and mechanical procedures, followed by centrifugation).

Recently, due both to the tendency of MSCs to progress into a hypertrophic phenotype [73] and to issues related to expansion, the concentrates’ regenerative potential has been explored. The idea is to transfer the entire regenerative potential present in the bone marrow or adipose tissue “stem cells niche” to the lesion site [74]. The most utilized approaches in orthopaedic clinical practice include the use of bone marrow aspirate (BMC) and stromal vascular fraction (SVF) from adipose tissue which comprise stem cells, growth factors, and ECM. They can be obtained with minimal manipulation in the operating room, without the need to isolate and expand the cells, and immediately implanted in the patient (Figure 3) [75]. Concentrates can be used alone or, frequently, with adjuvants, like platelet rich plasma (PRP) (see next paragraph), making evaluations more difficult, since there is not enough information on the regenerative effects of pure BMC or SVF [76]. Generally, clinical trials on OA patients revealed to be safe and reported some improvements in terms of cartilage regeneration and functional activity. However, the scarcity of long-term results and evidence suggest more in-depth studies to further confirm the efficacy of these kinds of treatment. [77,78,79].

### 3.3. Growth Factors

In articular cartilage, numerous growth factors (anabolic and catabolic) work synergically to regulate tissue development and homeostasis, increase cell growth and trigger differentiation. On this basis, they can be utilized as adjuvant favouring cartilage regeneration. Clinically available growth factors can be of synthetic or natural origin [24].

Synthetic growth factors recombinant polypeptides are: transforming growth factor beta (TGF-β1), bone morphogenetic protein (BMP)-2 and BMP-7 (also known as osteogenic protein-1, OP-1; each of the TGF-β superfamily); insulin-like growth factor-I (IGF-1); fibroblast growth factor-2 (FGF-2) and fibroblast growth factor-18 (FGF-18) of the fibroblast growth factor family and platelet-derived growth factor (PDGF) [80]. Several recent reports have indicated that the granulocyte-colony stimulating factor (G-CSF) serves not only as a growth factor for haematopoietic stem cells, but is also able to promote MSC mobilisation to both bone marrow and peripheral blood [81]. This discovery has been put into practice by Garay-Mendoza and colleagues, who conducted a prospective open-label, phase I/II clinical trial in patients with knee OA. Autologous bone marrow MSCs stimulated in vivo with G-CSF (subcutaneous administration), were administered through a single knee intra-articular injection. VAS and WOMAC scores showed significant improvement in knee pain and quality of life during a 6-month follow-up compared to the control group that received exclusively oral acetaminophen [82].

Among natural growth factors, to date, the most investigated is PRP. It is defined as a fraction of blood having a platelet concentration above baseline, obtained through density gradient centrifugation [75]. Platelets contain many important bioactive proteins and growth factors stored in their α-granules, including TGF-β1, PDGF, hepatocyte growth factor (HGF), b-FGF, epidermal growth factor (EGF), VEGF and IGF-I. All these growth factors are known to have a positive effect on tissue healing and regeneration [24]. The clinical use of PRP has been recently reviewed by Southworth et al., and by Mehranfar et al., who observed that a few clinical trials are generally available, and mostly consist in case studies. Moreover, they showed heterogeneity in terms of methods preparation, dosage and number of injections [76,83]. In some studies, PRP has been utilized as main treatment, as in the work by Sánchez et al., where severe knee OA was treated by combining intra-articular injections and intra-osseous infiltrations of PRP, with the aim to tackle several joint tissues simultaneously. The knee injury and osteoarthritis outcome score (KOOS) highlighted improved joint function and reduced pain. Moreover, PRP OA knee injections showed better results in terms of pain, functionality, and quality of the regenerated tissue in comparison with HA or corticosteroid injections or other treatments like prolotherapy [84]. In most studies, PRP is administered in aassociation with MSCs/ASCs or BMC, in order to potentiate tissue regeneration. In particular, in the work by Mayoly et al., 3 patients with OA were treated with a mixture of microfat (obtained from the subcutaneous adipose area of the inner side of the knee) and PRP. The only side effect was pain at the adipose tissue harvesting sites. Pain decrease of over 50% (per VAS score) was observed [75]. However, Bastos et al. found that adding PRP to OA knee MSCs injections did not provide additional benefits [85]. Similar results were obtained by the group of Koh by using ASCs from the infrapatellar fat, arthroscopic debridement, and PRP percutaneously injected into OA knees. Results were very close to the control group (injection without MSCs) [86]. Shapiro et al. conducted a single-blind, placebo-controlled trial on patients with bilateral knee OA, randomized to receive intra-articular injection of BMC combined with PRP into one knee and placebo into the other. The OARSI intermittent and constant osteoarthritis pain and VAS pain scores in both knees decreased significantly from baseline; pain relief did not differ significantly [87].

### 3.4. Gene Therapy

In the context of cartilage diseases, gene transfer has been performed in two ways: (i) in vivo injection/intravenous administration into the joint; and (ii) cell harvesting from the patient, ex vivo exposition to the vector and returning of the modified cells to the joint. Both methods can be associated with the use of a scaffold [33,34]. For example, chitosan has been utilized as a basis to entrap plasmids expressing genes involved in cartilage regeneration in chondrocytes [88,89]. Other studies have been also performed with MSC, and bone marrow concentrates [89]. Gene therapies with genes encoding for chondrogenic growth factors and anti-inflammatory cytokines are of interest to treat OA. In particular, in 2017, an ex vivo TGF-β1 gene therapy via retrovirus on allogeneic chondrocytes was approved [33]; in 2018, Kim et al. reported a study on the clinical efficacy of TissueGene-C (TG-C), a cell and gene therapy for knee OA in humans consisting of non-transformed and transduced chondrocytes (3:1), retrovirally transduced to overexpress TGF-β1. TG-C was associated with statistically significant improvements in function and pain in patients with knee OA [90].

The main limiting factors of gene therapy are the high costs, the transitory expression of the gene products and potential adverse effects, particularly in regards to carcinogenesis or the development of leukaemia by the use of viral vectors. In fact, although joint disorders are very common, often chronic, and frequently a source of considerable disability, their non-lethal nature raises the question of the benefit–risk ratio [34].

## 4. Future Perspectives

Regenerative medicine by the use of cell and gene therapy and tissue engineering has emerged as one of the most exciting areas of biotechnology, raising a plethora of hopes for researchers, clinicians, and patients. Having as its main purpose the regeneration of injured tissue and not its replacement with an external device, advances in this field may avoid treatments based on grafts and artificial prostheses. Moreover, regenerative medicine offers the possibility of personalization and to treat multi-factorial diseases such as OA.

However, while several studies appear to confirm that treatments are safe and efficacious, there are many limitations [91,92]. Generally, the trials are not uniform in terms of patient population, cell source, cell harvest, cell manipulation and number, treatment delivery, time elapsed at follow up, and the approaches utilized to evaluate improvement. This impairs clinical implementation and diffusion of regenerative therapies [93]. The question that arises is if regenerative medicine is able to provide an alternative to more conventional therapies [94,95]. Therefore, in the future, the conduction of more complete trials will be needed, in terms of large-scale and long-term follow-up and comparison with the control group.

The main issue in cartilage restoring therapies is the quality of the regenerated tissue, that should resemble as close as possible the native hyaline type. The ideal treatment would repopulate a lesion with chondrocytes able to produce a hyaline matrix, thus restoring cartilage structural and functional properties. In particular, the goal of reaching hyaline cartilage features is important to improve joint mechanics and thus delay OA progression. In this perspective, we identified four possible future perspectives for OA cartilage regeneration: pharmacological targeting, gene therapy, additive manufacturing and articular cartilage resident stem cells.

Pharmacological targeting is intended as the ability of a drug to accumulate in the target joint tissue. However, in many cases, a rapid drug clearance has been shown, minimally affecting the course of joint disease. To overcome this issue, numerous natural and synthetic scaffolds are being evaluated to achieve increased articular dwell, such as nanoscale carriers (for example, amphiphilic polymeric micelles) and hydrogels [96]. The targeting of native stem cells in order to restore initial tissue features (regeneration) has recently gained attention, especially due to the latest discoveries on the identification and characterization of “stem cell niches” in the joint [51,52].

Gene therapy has the potential to play an important and prominent part in regenerative medicine in the decades to come, although research is still needed [97]. Among recent research advancements, Lee et al. highlighted the importance of gene editing and, in particular of the clustered regularly interspaced short palindromic repeats (CRISPR) and CRISPR-associated (Cas)9 technology. CRISPR and Cas genes play an essential role in adaptive immunity in some bacteria, allowing the elimination of invading genetic materials. The CRISPR/Cas9 system has been already used in many studies to modify MSCs and chondrocytes with lentivirus-carried CRISPR/Cas9 [97], posing the basis for future use in orthopaedics. Rai et al. explored the potential of microRNAs (miRNAs) delivery on OA cartilage. Emerging options include the use of lipid nanoparticles or liposomes as carriers facilitating miRNAs transportation. Recent studies have concerned the development of miRNAs lentivirus-mediated injections [96].

**Figure 4:**
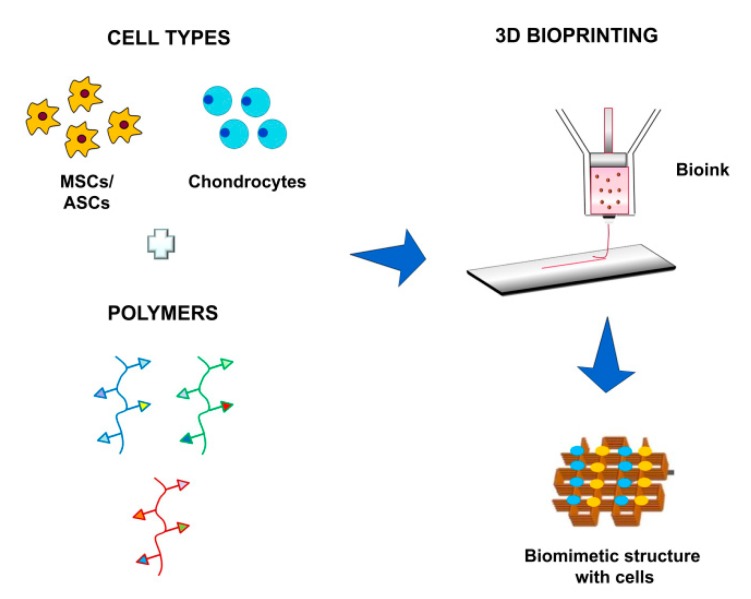
Schematic representation of the bioprinting process. Cells and polymeric scaffolds are mixed together (bioink) and dispensed on a substrate in a layer-by-layer approach, in order to build a living structure mimicking native tissue.

Additive manufacturing, also known as 3D printing or rapid prototyping, allows the fabrication of 3D scaffolds (computer-aided manufacturing, CAD), starting from computer-aided design (CAD) models obtained, for example, from MRI scans of patient lesions. It is usually realized in a layer-by-layer approach, as opposed to traditional subtractive methods where the material is removed from a starting piece to obtain the final product [98]. Additive manufacturing ensures more precision in object processing, allowing a better ability to mimic the structural, functional, and mechanical characteristics of native cartilage tissue (Figure 2). The possibility of creatinf scaffolds starting from the patient’s medical images, so as to match the size and shape of cartilage lesions, i.e., to tailor therapy, is promising for better addressing cartilage lesion repair [99]. Recently, the development of bioprinting technology allowed the deposit of layers composed of cells and polymeric materials (the combination often referred to as bioink), in order to form living tissues to be implanted with better possibility for successful treatment [100] (Figure 4). We believe that the bioprinting approach will allow a better mimic of hyaline cartilage architecture, facilitating advances in our endeavour to achieve optimal regeneration.

Recent studies conducted on animal models suggest the presence of native cartilage-resident stem/progenitor cells able to undergo chondrocyte differentiation during childhood and adulthood [101]. In particular, a type of stem cell has been identified in the resting zone of mouse growth plate which continues to generate columns of chondrocytes into adulthood [102]. Kozhemyakina et al. utilized an adult mouse model to knock the CreERT2-expressing cassette (a tamoxifen-inducible Cre allele) into Prg4 (Prg4GFPCreERt2) and to trace the progeny of Prg4+ cells at various ages. Prg4 encodes proteoglycan 4 (PRG4), also known as lubricin, which is expressed by cells at cartilage surface with the pivotal role of ensuring joint lubrication. They found that Prg4+ cells were located at the joint surface in the embryo as progenitors for deeper layers of mature cartilage. Comparably, Prg4GFPCreERt2 was expressed by superficial chondrocytes in young mice, and expanded into deeper regions with ageing [103]. Similar studies were performed on two other mouse strains and on juvenile animals, observing that the progeny of PRG4+ cells facilitated both appositional and interstitial growth of articular cartilage and could entirely reconstitute adult tissue [104,105,106]. Further research in mouse highlighted that Prg4+ cells are likely to be descendants of growth/differentiation factor 5 positive (GDF5+) cells. GDF5, expressed during limb development and before joint cavitation, is a member of the TGF-β superfamily which plays critical roles in cartilage morphogenesis [107]. Roelofs et al. showed that GDF5-traced cells are involved in the healing of cartilage defects. These findings on progenitor cells in animal articular cartilage have prompted investigation on their role in OA development and tissue regeneration in humans [108]. Rhee and colleagues showed that human patients and mice lacking lubricin develop non-inflammatory arthritis, postulating that lubricin has a role in diminishing the number of stem cells in cartilage, which is thus reduced in its regenerative ability upon damage [109]. Cartilage-resident MSC-like progenitor cells were also identified in cartilage from patients with OA: an early senescent phenotype was observed in one sub-population, possibly reflecting attempts to repair cartilage [107]. Evidence on cartilage-resident MSCs, together with data demonstrating that other stem cells populations are diffused in the whole joint (synovium, adipose tissue, and synovial fluid) with an involvement in the repair process, suggest a possible use for the treatment of chondral defects in patients with OA.

## 5. Conclusions

With the ageing of the population and rising obesity, OA has become a major problem for health systems globally. The regenerative approaches for the treatment of OA are growing and showing increasing potential in terms of safety, pain relief, improved function, and neo-cartilage formation. However, knowledge gaps and open questions still exist, particularly concerning heterogeneity of the treatments, number of patients, and long-term results. It is, therefore, recommendable to control treatment parameters, undertake large-scale clinical trials with long-term follow-up and evaluate improvements respect to conventional treatments.
